# New Appliances and Things Medical

**Published:** 1900-07-07

**Authors:** 


### NEW APPLIANCES AND THINGS MEDICAL.

**[We shall be glad to receive, at our Office, 28 & 29, Southampton Street, Strand, London, W.C., from the manufacturers, specimens of all new preparations and appliances which may be brought out from time to time.]**

### IZAL PREPARATIONS.

(Newton, Chambers, and Co., Ltd., Thorncliffe, Sheffield.)

In a recent number of this journal we inadvertently referred to Izal as an emulsion of coal tar acid of the phenol type. Our attention has been drawn to the fact that by an ingenious method of preparation the group of bodies to which Izal owes its remarkable antiseptic powers are derived from direct distillation of coal without the intermediate production of tar. The bodies thus formed present chemical properties of extreme interest, and constitute a group which possess antiseptic properties of the highest order, which although closely allied to the phenol group are distinct and separate chemical entities.

### RED WINE FOR INVALIDS.—RIOGA BRILLANTE PALOMA.

(P. De Otaduy and Co., 20, High Holborn, London, W.C.)

This excellent Spanish wine should be well suited to the requirements of convalescent invalids who desire a wine of somewhat generous character and yet free from excess of sugar, such as detracts from the value of wine of the nature of port under similar circumstances. Paloma is a red wine of great purity, and, as analysis shows, contains a comparatively low percentage of volatile free acids (·92 per cent.) Its alcoholic strength, by weight, is rather under 10 per cent., and its organic solids about 1·46 per cent. The pleasant flavour of the wine and its sustaining qualities, combined with great moderation in price, should commend it to many doctors and invalids who are in search of wines of this stamp.

